# Family Caregivers on the Frontline: Challenges of Providing Care to Post-ICU COVID-19 Patients

**DOI:** 10.1093/geroni/igaa057.3463

**Published:** 2020-12-16

**Authors:** Amanda Leggett, Alicia Carmichael, Natalie Leonard, Sheria Robinson-Lane, Sophia Li, Grace Oxford, Maren Wisniewski, Richard Gonzalez

**Affiliations:** 1 The University of Michigan, Ypsilanti, Michigan, United States; 2 University of Michigan, Ann Arbor, Michigan, United States

## Abstract

Family caregivers are essential care providers helping to ensure the sometimes complicated recovery of recently hospitalized COVID-19 patients. COVID-19 caregivers face pandemic-specific challenges such as not being at patient bedside throughout the hospital stay and managing social distancing post-discharge. The current study aims to explore the unique experiences of family caregivers of Intensive Care Unit (ICU) COVID-19 patients. In-depth qualitative interviews were conducted by web conference with 13 dyads of adults who were in an ICU for COVID-19 between March and August 2020 and their primary caregiver (n=26). Participants were interviewed about the care recipient’s hospitalization and recovery journey, supports received, challenges experienced, and gaps in the system of care. Thematic qualitative analysis was conducted utilizing Watkins’ (2017) rigorous and accelerated data reduction (RADaR) technique. Caregivers played a critical role in patient admission, discharge, and recovery. Themes of caregiving challenges included self-management of COVID-19 infection, knowledge deficits of available resources and post-discharge care needs, post-infection stigma, separation guilt, deprioritized self-care, financial challenges, and lengthy recoveries with some ongoing health needs. While receipt of emotional support was considered an advantage, some caregivers expressed contact fatigue. Understanding how COVID caregivers experience illness management across the recovery journey can aid our understanding of the COVID caregiving process and identify intervention targets to improve overall health and well-being of the care dyad.

